# Carotenoids Assist in Cyanobacterial Photosystem II Assembly and Function

**DOI:** 10.3389/fpls.2016.00295

**Published:** 2016-03-10

**Authors:** Tomas Zakar, Hajnalka Laczko-Dobos, Tunde N. Toth, Zoltan Gombos

**Affiliations:** Laboratory of Plant Lipid Function and Structure, Institute of Plant Biology, Biological Research Centre, Hungarian Academy of SciencesSzeged, Hungary

**Keywords:** carotenoids, Photosystem II, cyanobacteria, *Synechocystis*, phycobilisomes, xanthophylls

## Abstract

Carotenoids (carotenes and xanthophylls) are ubiquitous constituents of living organisms. They are protective agents against oxidative stresses and serve as modulators of membrane microviscosity. As antioxidants they can protect photosynthetic organisms from free radicals like reactive oxygen species that originate from water splitting, the first step of photosynthesis. We summarize the structural and functional roles of carotenoids in connection with cyanobacterial Photosystem II. Although carotenoids are hydrophobic molecules, their complexes with proteins also allow cytoplasmic localization. In cyanobacterial cells such complexes are called orange carotenoid proteins, and they protect Photosystem II and Photosystem I by preventing their overexcitation through phycobilisomes (PBS). Recently it has been observed that carotenoids are not only required for the proper functioning, but also for the structural stability of PBSs.

## Roles of Carotenoids in Modulating Photosynthetic and Cytoplasmic Membranes of Cyanobacteria

### Carotenoids as Protective Agents

Carotenoids are a group of pigments that play multiple structural and functional roles. They are molecules with conjugated double bonds, which allow them to participate in photosynthetic functions (Gruszecki and Strzalka, 2005). In cyanobacteria the most abundant carotenoids are carotenes (e.g., β-carotene) and various types of xanthophylls (synechoxanthin, canthaxanthin, caloxanthin, echinenone, myxoxanthophyll, nostoxanthin, zeaxanthin), which are oxygenated derivates of the carotenes (Takaichi and Mochimaru, 2007; Domonkos et al., 2013; Kusama et al., 2015; Toth et al., 2015).

Carotene and xanthophyll molecules are indispensable components of both cytoplasmic and thylakoidal membranes of cyanobacteria. However, β-carotene is the only carotenoid, which was localized in PSII by X-ray crystallography (Loll et al., 2005; Umena et al., 2011). As an extremly hydrophobic molecule, β-carotene forms complexes with proteins, thereby functioning as a bridge between various proteins involved in photosynthetic processes. In thylakoid membranes a protective role of β-carotene was demonstrated as a scavenger of singlet oxygen (Packer et al., 1981).

In contrast to β-carotene, xanthophylls have not been localized unambiguously. Their presence in the photosystems is expected, and has been demonstrated by various biophysical and biochemical methods (Van der Weij-de Wit et al., 2007; Domonkos et al., 2009). Nevertheless the binding of xanthophylls to membrane proteins has not yet been demonstrated. In the membranes they are positioned in a way that their hydrophilic part faces the aqueous phase.

Free carotenes and xanthophylls occupy the hydrophobic region of membrane bilayers. Accordingly, it was predicted that xanthophylls rigidify membranes, whereas β-carotene and echinenon have a neutral or fluidizing effect (Gruszecki and Strzalka, 2005).

Although the scavenging character of xanthophylls is stronger than that of β-carotene (Steiger et al., 1999; Domonkos et al., 2013), neither their localization nor their exact scavenging role has been elucidated yet. Xanthophylls are found mainly in cytoplasmic membranes (Masamoto and Furukawa, 1997; Masamoto et al., 1999). A recent review has summarized the protective mechanism of carotenoids and other putative protective functions against ROS, which are produced by PSII of photosynthesis that mediates light-driven oxidation of water and the release of molecular oxygen (Derks et al., 2015).

### Carotenes and Xanthophylls Affect Membrane Viscosity

Due to their influence on membrane properties, the xanthopylls are also important in the adaptation to various temperature conditions. Low growth temperatures induce enhanced synthesis of xanthophylls and, consequently, the xanthophyll content of thylakoid membranes is increased to compensate for elevated lipid desaturation. Instead of increasing membrane microviscosity, enhanced xanthophyll content alters membrane dynamics by allowing a tighter arrangement of fatty acyl moieties. The observed discrepancy could be explained by an apparent increase of very rigid, myxoxanthophyll-related lipids in the thylakoid membranes (Varkonyi et al., 2002).

### Presence of Enzymes of Carotenoid Biosynthesis in Cytoplasmic Membrane

More recent research has shown that cytoplasmic membranes contain some biosynthetic enzymes specific for the biosynthesis of echinenone and β-carotene. The CrtQ (zeta-carotene desaturase) and CrtO (beta-carotene ketolase) enzymes involved in carotenoid synthesis are localized in cytoplasmic membranes (Zhang et al., 2015). This would mean that echinenone and precursors of β-carotene are more abundant in cytoplasmic membranes than in thylakoids. Cytoplasmic membranes contain higher amounts of carotenoids than thylakoid membranes, which results in a more pronounced modulation of their membrane fluidity.

Despite the relatively low carotenoid content of the thylakoid membrane, these compounds are indispensable for photosynthetic processes. They not only affect the membrane structure, but also influence the oligomerization of proteins, thereby modulating photosynthetic functions.

## The Effect of Carotenoids on the Architecture and Various Functions of Photosystem II Complexes

### Structure

X-ray crystallography revealed that in thylakoid membranes the main carotene (β-carotene) localizes in the photosynthetic reaction centers (Loll et al., 2005; Umena et al., 2011). These results revealed that a PSII complex contains 11 β-carotene molecules. Xanthophylls could not be localized unambiguously. The importance of carotenoids in maintaining the stability and functioning of PSII was demonstrated in a carotenoid-deficient mutant strain (Sozer et al., 2010).

The completely carotenoid deficient Δ*crtH/B* or Δ*crtB* mutants of *Synechocystis* sp. PCC 6803 (hereafter *Synechocystis*) are devoid of active PSII reaction centers. These strains do not show oxygen-evolving activity (Sozer et al., 2010; Toth et al., 2015). The cells have reduced translation for a number of genes encoding PSII proteins and hence do not assemble active PSII complexes. They are capable of synthesizing only a limited amount of CP47, which is crucial for the formation of dimeric PSII complexes, but CP43 synthesis is almost completely blocked. The lack of these protein subunits prevents the formation of functional PSII complexes. This is the reason why we think that carotenoids are indispensable components of PSII.

**FIGURE 1:**
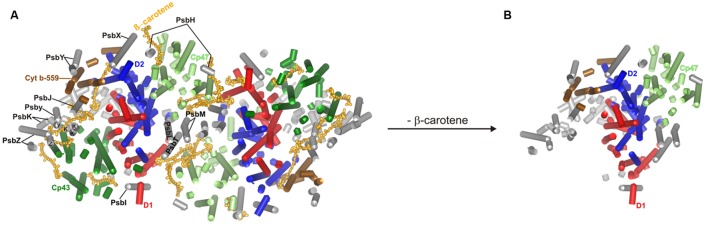
**Structure of the PSII complex deduced from X-ray crystallographic data (PDB files: 3BZ1 and 3BZ2)**. The PSII supercomplex in wild type **(A)** and assumed PSII structure in carotenoid-deficient **(B)** *Thermosynechococcus elongatus*.

Complete loss of carotenoids prevents the proper assembly of PSII (**Figure 1**), and such cells contain almost exclusively CP43-less oligomers. They show reduced PSII fluorescence, which originates from PSII components that are unable to integrate into a fully functional complex. In the carotenoid-less mutant Δ*crtB* we observed the accumulation of fluorescence-emitting spots with long lifetime close to the cell walls (Toth et al., 2015, Supplementary Figures). This could indicate that PSII biogenesis is blocked and incomplete adducts remain localized at the thylakoid-organizing complexes. This is in good agreement with an earlier observation of dysfunctional thylakoid membrane systems in a carotenoid-deficient mutant (Nickelsen et al., 2011). The PSII structure of *Thermosynechococcus elongatus*, deduced from X-ray crystallographic data (Guskov et al., 2009) shows the presence of various protein subunits and cofactors, such as carotenoids, in the dimeric form of reaction centers (**Figure 1A**). The structure of the imagined carotenoid-less PSII is presented in **Figure 1B**. However, the reaction center in Δ*crtB* cells contains almost exclusively CP43-less oligomers. Radioactive labeling demonstrated limited synthesis of inner PSII antennae, CP47 and, particularly, CP43. The lack of CP43 resulted in the formation of the RC47 pre-assembly complex (Komenda et al., 2012), demonstrating that carotenoid deficiency results in low levels of partially assembled reaction center complexes. These cells show reduced fluorescence originating from the remaining chlorophyll-protein complexes that are unable to integrate into a complex (Sozer et al., 2010; Toth et al., 2015).

Carotenoids, together with chlorophylls, are also necessary for the translation and stabilization of photosynthetic reaction center apoproteins in the green alga *Chlamydomonas reinhardtii*, however, they do not regulate the transcription of reaction center apoproteins (Herrin et al., 1992).

Our recent results suggest some structural function of xanthophylls in stabilizing PSII, as well as its dimeric complexes in *Synechocystis* (Toth et al., 2015). Carotenoids are essential mediators of interactions between PSII monomers, highlighting the role of neutral lipids, which can regulate the equilibrium between dimerization and dissociation (Guskov et al., 2009).

Accordingly, carotenoids are indispensable constituents of the photosynthetic apparatus, being essential not only for antioxidative protection but also for the productive synthesis and accumulation of photosynthetic proteins and, especially, those of the PSII antenna subunits.

### Function

Carotenoids, due to their light harvesting (Stamatakis et al., 2014) and photoprotective capacity (Schafer et al., 2005; Sozer et al., 2010), are indispensable for the function of the photosynthetic apparatus, and particularly in that of PSII. Under low light conditions carotenoids can provide more efficient light absorption (Koyama et al., 1996; Bode et al., 2009). By contrast, when cyanobacteria are exposed to high light, excess energy needs to be reduced to avoid photoinhibition damage to the photosystems (Powles, 1984; Aro et al., 1993, 2005). Carotenoids can exert protection by dissipating excess energy as heat, a phenomenon called NPQ, or by scavenging ROS.

The activity of light-damaged PSII is efficiently restored by a repair system, therefore photoinhibition occurs only when the rate of inactivation exceeds that of repair. In order to understand the mechanism by which carotenoids protect the cells under light stress it would be necessary to study the two processes of photoinhibition, photodamage and repair, separately (Nishiyama and Murata, 2014).

Photosystem II complexes are very sensitive not only to strong light but also to a wide range of abiotic stress effects, such as low and high temperatures, UV-B exposure, drought, and high concentration of salts (Takahashi and Murata, 2008). These stress factors ultimately lead to oxidative stress. Recently it has been shown that Δ*sigCDE*, a group 2 σ factor mutant of *Synechocystis*, is more sensitive to oxidative stress, but also more resistant to the photoinhibition of PSII. In this mutant an up-regulation of photoprotective carotenoids was observed, but it has been suggested that the resistance to light damage of PSII and the overall tolerance to oxidative stress are distinct in cyanobacteria, and their mechanisms are different (Hakkila et al., 2014).

In order to study the specific role of different carotenoids in cyanobacteria a wide range of carotenoid-deficient mutants were generated. Studies with various xanthophyll mutants of *Synechococcus* sp. PCC 7002 indicate that xanthophylls contribute to protection against photo-oxidative stress (Zhu et al., 2010). Similarly *Synechocystis* mutants lacking almost all xanthophylls (Δ*crtRO*) were sensitized to photodamage only under high light conditions (Schafer et al., 2005), whereas under normal illumination charge separation in PSII seemed unaffected (Toth et al., 2015). Recently it has been shown that zeaxanthin and echinenon protect the repair part of the PSII recovery cycle from photoinhibition by decreasing the level of singlet oxygen that inhibits protein synthesis (Kusama et al., 2015).

The removal of all β-carotene and xanthophylls from *Synechocystis* (Δ*crtH/B* and Δ*crtB* mutants) causes more severe effects. These strains are extremely light sensitive and they can grow only in the dark under light-activated heterotrophic conditions, without detectable oxygen evolution (Sozer et al., 2010). Time-resolved fluorescence (streak camera) measurements of these mutant cells also indicate inactive PSII (Toth et al., 2015).

In PSII reaction centers there are at least two redox active β-carotenes (Telfer et al., 2003). Recently it has been shown that one of the two so-called redox active carotenoids, Car_D2_, plays a role in photoprotection. Site-directed mutations around the binding pocket of Car_D2_ in *Synechocystis* cells revealed the importance of β-carotenes in the initiation of secondary electron transfer processes, which occurs when water oxidation is inhibited (Shinopoulos et al., 2014).

In addition to the role of carotenoids as constituents of membrane-embedded PSII, they also have crucial functions in the formation of protein complexes, the so-called photoactive orange carotenoid proteins. A recent study has elucidated the dual role of carotenoids in OCP, namely the protection of photosystems by quenching excess energy, and also the capacity of quenching singlet oxygen formed during the light reactions (Sedoud et al., 2014). Using an OCP-deficient mutant it has been shown that OCP-related thermal dissipation protects the repair of PSII during photoinhibition (Kusama et al., 2015). Although it is clear that carotenoids are the principal actors in energy dissipation, the mechanism of this process is poorly understood. Staleva et al. (2015) pointed out a new function of carotenoids associated with proteins. They have shown that *Synechocystis* HliD, a Hlips (high light-inducible proteins) family protein, binds chlorophyll *a* and β-carotene with a 3:1 ratio. The photoprotective role of this carotenoid-binding protein was demonstrated by femtosecond spectroscopy, showing that energy dissipation is achieved via direct energy transfer from a chlorophyll *a* Qy state to the β-carotene S1 state (Staleva et al., 2015).

Currently global changes in the environment can generate a multitude of stress factors, such as high or low temperatures, high light or UV-B radiation, etc. This underlines the importance of the evolutionary role of carotenoids and the mechanisms by which they can contribute to the survival of various organisms under extreme conditions.

## How do Carotenoids Affect the Structure and Processes of Phycobilisomes of Cyanobacterial Photosystem II Complexes?

### Structure

Cyanobacteria have special light-harvesting complexes, PBSs, which can absorb light in a wide spectral range. In addition to PBS, carotenoids can function as accessory pigments to widen the range of absorption. PBSs are comprised of rods attached to a core complex that is directly linked to PSII. In *Synechocystis* the rods contain phycocyanin, which can harvest long wavelength light and transfer its energy to the allophycocynin core, which then transduces it directly to the PSII reaction center (Maccoll and Guard-Friar, 1986). In the reaction center the main pigment is chlorophyll *a*, whereas in the PBS the chromophore is phycocyanobilin or some other, structurally related pigment.

**FIGURE 2:**
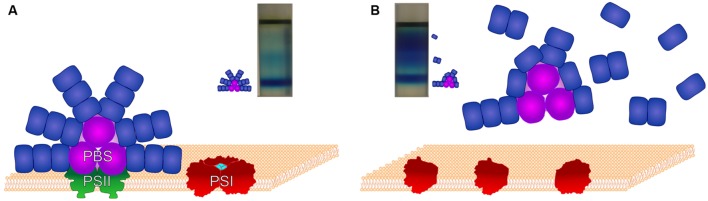
**The structures of PBS and the photosynthetic reaction centers in wild-type *Synechocystis* and its Δ*crtB* carotenoid-less mutant**. Schematic figure showing the assembled supercomplexes and the positions of their constituents following separation by stepwise sucrose density gradient centrifugation. **(A)**: Fully assembled functional supercomplexes of wild-type cells with entire PBS, dimeric PSII and trimerized PSI. **(B)**: Monomeric PSI and partially assembled PBS of the Δ*crtB* mutant. In these cells only a negligible amount of PSII is formed.

In the PBS structure the presence of β-carotene or xanthophylls has not yet been demonstrated. Surprisingly, in carotenoid-less Δ*crtB* cells a large amount of unconnected phycocyanin units has been observed (Toth et al., 2015). Density gradient centrifugation revealed that most of the assembled core complexes had shorter rods than those of the wild type. In this mutant PSI is in monomeric form, only a negligible amount of PSII is formed, and CP43 is mostly detached from PSII. Schematic structures of the photosynthetic complexes, as well as images of the sucrose density gradient-separated PBS components of wild-type *Synechocystis* and its carotenoid-less Δ*crtB* mutant are shown in **Figure 2**.

It seems that carotenoids are required for the assembly or maintenance of the complete PBS structure (Toth et al., 2015), suggesting a direct or indirect effect of carotenoids on the structure and functions of this complex (Toth et al., 2015).

### Light-Harvesting Process

Cyanobacterial carotenoid-proteins play an important role in photoprotection (Kerfeld et al., 2003; Kerfeld, 2004). One of these, the water-soluble OCP, has been structurally characterized and has recently emerged as a key player in cyanobacterial photoprotection (Sedoud et al., 2014). OCP was first described by David Krogmann more than 25 years ago (Holt and Krogmann, 1981). Highly conserved homologs of the gene encoding the 34 kDa OCP are present in most of the known cyanobacterial genomes.

The carotenoid composition of the OCP isolated from wild-type cells is as follows: 60% echinenone, 30% keto-carotenoid 3′-hydroxyechinenone, and 10% zeaxanthin (Sedoud et al., 2014). The energy collected by the PBS is rapidly transferred from rods to the core, and subsequently to membrane-embedded reaction centers of PSII or PSI.

Photoactive orange carotenoid protein is a photoactive protein. Illumination of OCP by strong blue-green light induces changes in its carotenoid, converting the inactive orange dark form (OCPo) into an active red form (OCPr). In OCPo, 3′-hydroxyechinenone is in all-trans configuration. In OCPr, it is also in all-trans configuration, but its apparent conjugation length increases, resulting in a less distorted, more planar structure. Fourier transform infrared spectra showed that these changes in the carotenoid induce conformational changes in the protein, leading to a less rigid helical structure and a compaction of the β-sheet. These changes in OCP are essential for the induction of the photoprotective mechanism. Only OCPr is capable of binding to the PBSs, inducing fluorescence quenching and the photoprotective mechanism (Wilson et al., 2012).

In contrast to photosynthetic eukaryotes, photoprotection in cyanobacteria is not induced by transthylakoid ΔpH or excitation pressure on PSII. Instead, intense blue–green light (400–550 nm) induces quenching of PSII fluorescence that is reversible in minutes, even in the presence of translation inhibitors (El Bissati et al., 2000). Fluorescence spectra and the study of NPQ mechanism in PBS- and PSII-mutants of *Synechocystis* indicate that this mechanism involves a specific decrease in the fluorescence emission of PBSs, as well as a decrease of energy transfer from PBS to the reaction centers (Scott et al., 2006; Wilson et al., 2006). The site of the quenching appears to be the core of the PBS (Scott et al., 2006; Wilson et al., 2006; Rakhimberdieva et al., 2007).

The action spectrum of PBS fluorescence quenching resembles the absorption spectrum of cyanobacterial carotenoids. In the absence of OCP, strong white or blue–green light-induced NPQ was completely inhibited in *Synechocystis*. As a consequence, OCP-deficient cells are more sensitive to light stress. Moreover, the action spectrum of cyanobacterial NPQ (Rakhimberdieva et al., 2004) exactly matches the absorption spectrum of the carotenoid, 3′-hydroxyechinenone (Polivka et al., 2005) in the OCP. OCP is now known to be specifically involved in the PBS -associated NPQ and not in the other mechanisms affecting the levels of fluorescence, such as state transitions or D1 damage (Wilson et al., 2006; Zhang et al., 2014). Electron microscopic studies using immunogold labeling revealed that the majority of OCP is localized in the inter-thylakoidal cytoplasmic region, on the PBS side of the membrane (Wilson et al., 2006). The interaction between the OCP and the PBSs and thylakoids was corroborated by the presence of OCP in the PBS-associated membrane fraction (Wilson et al., 2006, 2007). In *Synechocystis*, OCP is constitutively expressed, and it is present even in mutants that lack PBSs (Wilson et al., 2007). Stress conditions (high light, salt stress, iron starvation) increase levels of the OCP transcript and proteins (Hihara et al., 2001; Kanesaki et al., 2002; Fulda et al., 2006; Wilson et al., 2007). All known OCP-like genes of cyanobacteria are transcriptionally active and the NPQ mechanism is inducible by blue light. This suggests that the OCP-based photoprotective mechanism is widespread in cyanobacteria (Boulay et al., 2008).

Fluorescence recovery protein is known to be involved in photoprotection and restoration of full light-harvesting capacity. FRP is needed for the recovery of full antenna capacity when light intensity decreases (Boulay et al., 2010). FRP interacts with the activated OCP and accelerates its deactivation and detachment from the PBS (Boulay et al., 2010; Gwizdala et al., 2011), although the details of these processes have not yet been elucidated (Zhang et al., 2014).

Carotenoid containing OCP molecules have an essential role in cyanobacterial photoprotection by binding to the PBS.

## Conclusion

Carotenoids are important for multiple PSII functions, as they are not only required for its activity but also participate in the light-harvesting process. They act as pigments, which can increase the spectral range, and can also protect against over-excitation and oxidative side products. It has been shown that β-carotene is essential for the assembly of PSII and the PSI trimer, whereas xanthophylls can stabilize them. The influence of OCP on the light-harvesting capacity of PBS and the requirement of carotenoids for the proper assembly and function of this complex highlight important additional roles of not only β-carotene, but also of the xanthophylls in cyanobacterial photosynthesis. Further studies are required to elucidate the exact mechanisms by which carotenoids influence the structure and function of PSII and the PBS, and how they contribute to the protection of the photosynthetic processes.

## Author Contributions

The authors of this mini-review equally contributed to its concept and design. The tasks were assigned as follows: TZ contributed to all chapters and had a key role in formulating the review. HL-D wrote a chapter, critically read and corrected the entire manuscript, and adjusted it to the required format. TT prepared the figures, the list of references, and gave advice on the text. ZG provided the general concept and design, and also contributed to a chapter.

## Conflict of Interest Statement

The authors declare that the research was conducted in the absence of any commercial or financial relationships that could be construed as a potential conflict of interest.

## ABBREVIATIONS

CP43: chlorophyll-protein complex 43
CP47: chlorophyll-protein complex 47
FRP: fluorescence recovery protein
NPQ: non-photochemical quenching
OCP: photoactive orange carotenoid protein
PBS: phycobilisome
PSI and PSII: Photosystem I and II
RC47: reaction center 47
ROS: reactive oxygen species
*Synechocystis*: *Synechocystis* sp. PCC6803.
